# Creation and Validation of the Major Pediatric Mitochondrial Cytopathies Minimum Data Set: Consensus from a Moroccan–Tunisian Delphi Study

**DOI:** 10.3390/children12091121

**Published:** 2025-08-26

**Authors:** Sara El Guessabi, Jihane Belayachi, Ilhem Ben Youssef Turki, Ichraf Kraoua, Said Galai, Hind Lachraf, Ilham Ratbi, Redouane Abouqal, Yamna Kriouile

**Affiliations:** 1 Laboratory of Genomics and Molecular Epidemiology of Genetic Diseases: Genes and Mutations in the Moroccan Population, Faculty of Medicine and Pharmacy of Rabat, Mohammed V University in Rabat, Rabat 10000, Morocco; 2 Laboratory of Biostatistics, Clinical Research and Epidemiology, Public Health Department, Faculty of Medicine and Pharmacy of Rabat, Mohammed V University in Rabat, Rabat 10000, Morocco; 3 LR18SP04, Department of Pediatric Neurology, National Institute of Neurology Mongi-Ben Hamida, Tunis El Manar University, Tunis 1007, Tunisia; 4 LR18SP04, Department of Clinical Biology, National Institute Mongi Ben Hmida of Neurology, Tunis 1007, Tunisia; 5 Department of Paediatrics 2, Unit of Endocrinology and Neuropediatrics, Children’s Hospital of Rabat, Rabat 6527, Morocco; 6 Department of Medical Genetics, National Institute of Health in Rabat, Rabat 10090, Morocco

**Keywords:** mitochondrial cytopathies, minimum data set, registry, pediatrics, Maghreb, Delphi study, consensus

## Abstract

**Highlights:**

**What are the main findings?**

- A Minimum Data Set (MDS) of 347 validated variables was developed for pediatric mitochondrial cytopathies through a binational Delphi process involving experts from Morocco and Tunisia.
- The MDS is structured into 5 sections, 21 categories, and 46 subcategories, covering clinical, biochemical, molecular, and therapeutic data.

**What is the implication of the main finding?**

- This MDS lays the foundation for a binational registry to improve diagnosis, care, and research on pediatric mitochondrial diseases in North Africa.
- The model supports future interoperability with international registries and enhances regional participation in multicenter studies and clinical trials.

**Abstract:**

**Background**: Pediatric mitochondrial cytopathies (MCs) are rare, multisystemic, and heterogeneous disorders that require harmonized collection of clinical, biochemical, and genetic data to better understand their natural history, optimize patient care, and support translational research. In this context, developing a regionally adapted Minimum Data Set (MDS) is a critical step toward establishing a structured registry. **Methods**: A two-round Delphi study was conducted involving 16 Moroccan–Tunisian experts from diverse specialties to assess the relevance of 382 initially proposed variables. Robust statistical analyses were applied to all composite questions using Content Validity Index (CVI), Kappa coefficient, and Content Validity Ratio (CVR), alongside retention rate assessments. **Results**: The overall relevance score assigned by the experts was high (4.5 ± 0.41), with a final retention rate of 90.1% (347 variables retained out of 382). Section-wise S-CVI/Ave scores ranged from 0.91 to 0.99, with the paraclinical section achieving the highest value (0.99) and the evolutive section the lowest (0.91). The more stringent S-CVI/UA revealed greater variability (from 0.36 in clinical data to 0.83 in paraclinical data). Kappa index calculations led to the exclusion of a subclass of five therapeutic variables due to insufficient inter-rater agreement. The CVR further supported the content validity of the 46 retained subclasses. The results demonstrated strong consensus, particularly across the neurological, biochemical, molecular, and medical follow-up domains. Additionally, the registry design survey revealed strong expert support for a secure and interoperable digital platform incorporating longitudinal follow-up and advanced search and reporting functionalities. **Conclusions**: This validated Moroccan–Tunisian pediatric MDS offers a solid foundation for a regional mitochondrial cytopathy registry. It standardizes data collection, strengthens clinical research, and improves diagnosis and care for affected children in the Maghreb. Moreover, it lays the groundwork for future interoperability with international registries, contributing to a more inclusive and collaborative precision medicine landscape.

## 1. Introduction

Mitochondrial cytopathies (MCs) are a heterogeneous group of rare genetic diseases characterized by dysfunction of the mitochondrial respiratory chain, which plays a crucial role in cellular energy production. These disorders represent one of the most frequently encountered groups among inborn errors of metabolism, with a global incidence estimated at approximately 1 in 4300 (2019). In pediatric populations, prevalence varies across studies, but is estimated to be around 1 in 11,000 to 1 in 20,000 (1999) for mitochondrial encephalomyopathies [1].

Due to defects in cytosolic energy transmission, MCs commonly affect organs with high energy demands such as the brain, heart, muscles, and liver. Their clinical expression is highly variable, ranging from early psychomotor delays to multifactorial multiorgan involvement [2]. These diseases are among the leading causes of morbidity and mortality in early childhood, even during the neonatal period [3,4]. Their rarity and wide spectrum of clinical presentations make diagnosis particularly difficult, often delaying medical intervention. Therapeutic options remain limited, and current management is primarily symptomatic, focusing on multidisciplinary care aimed at improving quality of life. Because of these features, MCs represent a major pediatric public health concern [1,5].

Despite their importance, MCs are still poorly studied from an epidemiological perspective, particularly in low-resource countries. The lack of structured registries in many of these settings, such as in Morocco and Tunisia, makes systematic data collection impossible, while clinical information often remains scattered across various healthcare centers and is inconsistent [6,7]. Consolidating this information would require the development of longitudinal or comparative studies, as conventional hospital databases are rarely suitable for describing the complex characteristics of rare diseases such as MCs [8,9,10].

A clinical registry is “a system for collecting information on patients with a specific disease, based on the use of standardized methods” (Agency for Healthcare Research and Quality, AHRQ) and can help improve the characterization of rare diseases, clinical practice, recruitment into therapeutic trials, and even inform public health policies [11]. In the case of MCs, developing a structured registry is essential to improving clinical follow-up, research, and treatment strategies [12,13].

The first step in making a useful registry is to define a Minimum Data Set (MDS), which is a standard set of important information that must be collected for each patient. The MDS makes sure that data entry is consistent, which makes it easier to compare data from different centers and over time [14]. In the field of rare diseases, where every piece of information is important, a strict MDS is a key tool for improving both research and clinical care [15].

The complexity of MCs, however, requires the collection of various types of data: evolving clinical signs, biochemical parameters such as lactate and acylcarnitines, enzymatic activities of respiratory chain complexes, as well as molecular findings from mitochondrial or nuclear DNA sequencing [16]. This multidimensional profile necessitates collaboration among multiple medical and scientific specialties, including neuropediatrics, genetics, biochemistry, and neurology [17].

Designing a specific MDS that incorporates this complexity is therefore essential to ensure data completeness, consistency, and usability [18].

The effort to create a pediatric MC registry is part of a joint Morocco–Tunisia research collaboration aimed at improving the diagnosis and monitoring capabilities in both countries. These rare, complicated, and diverse diseases continue to go unnoticed and undocumented in the Maghreb region. To date, there is no national or regional registry that collects clinical, biochemical, and molecular data on these illnesses. This absence is a significant barrier to understanding local epidemiology, identifying the most common mutations, and optimizing patient care.

Systematic registration of cases is thus essential to document field realities, harmonize practices across centers, and generate data that can be used for research, early diagnosis, and therapeutic evaluation. The development of a standardized MDS is one of the primary objectives of this project: to lay the foundations for a shared, structured, and sustainable registry for pediatric mitochondrial cytopathies within our regional context.

## 2. Materials and Methods

This methodological Delphi study was conducted in three successive and complementary phases aimed at developing and validating a Minimum Data Set (MDS) for pediatric mitochondrial cytopathies (MCs):

- A literature review was performed to identify relevant variables;
- A Delphi survey was conducted to assess the importance of each proposed item, as evaluated by a multidisciplinary panel of experts;
- A rigorous statistical assessment of the content validity of the MDS was carried out using recognized indicators.

The methodological framework followed international recommendations for the development of clinical information systems and the construction of MDSs, particularly those issued by the Health Information and Management Systems Society (HIMSS), which emphasize system design, data standardization, and interoperability. Additionally, the framework incorporated best practices reported in the scientific literature, including stakeholder engagement, the use of multiple validated content validity indices (I-CVI, S-CVI, Kappa, CVR), and iterative Delphi rounds to ensure the MDS’s relevance, feasibility, and adaptability to the specific clinical and resource contexts of pediatric mitochondrial cytopathies in the Maghreb region [19,20,21].

### 2.1. Literature Review

We conducted a narrative literature review to identify the most frequently reported clinical, biochemical, molecular, and administrative variables related to pediatric mitochondrial cytopathies (MCs). This review focused on variables describing neurological and developmental manifestations, biochemical markers such as respiratory chain enzyme activities, genetic mutations affecting mitochondrial DNA, and administrative data including demographic and follow-up information. The aim was to collect relevant data elements to guide the development of a Minimum Data Set (MDS) applicable to both clinical practice and epidemiological research in childhood mitochondrial diseases.

#### 2.1.1. Search Strategy

We searched multiple biomedical databases, primarily PubMed, Scopus, ScienceDirect, and the Google Scholar search engine, between December 2023 and May 2024, using the following keywords and Boolean operators:

(“mitochondrial cytopathy” OR “mitochondrial disease” OR MELAS OR “Leigh syndrome” OR LHON OR “Pearson syndrome”) AND (“data” OR “variables” OR “dataset” OR “data element” OR “clinical features” OR “biochemical markers” OR “molecular diagnosis” OR “minimum data set”) AND (child OR pediatric)

We included full-text articles published in English or French that focused on MCs in children or adolescents. Irrelevant documents such as editorials, letters to the editor, or abstracts lacking original data were excluded.

#### 2.1.2. Selection and Data Extraction

The selected publications were manually reviewed. Relevant information was extracted and categorized into four main domains:

- Administrative Data: Patient identifiers, family linkage, date of diagnosis, healthcare facility, country, and ethnic origin.
- Clinical Data: Initial symptoms (developmental delay, ataxia, hypotonia, epileptic seizures, etc.), age at onset, disease progression, and multiorgan involvement.
- Paraclinical Data: Including biochemical markers (lactate levels, pyruvate levels, enzymatic activity of respiratory chain complexes in muscle or fibroblasts) and molecular data (frequent mitochondrial DNA mutations such as m.3243A>G, m.8993T>G/C, results from mtDNA or nuclear gene panel sequencing, and deletion or duplication testing).
- Therapeutic and Follow-Up Data: “Treatments administered” encompassed pharmacological therapies, dietary interventions, supplementation with cofactors and vitamins, as well as supportive care measures. “Disease progression indicators” included relevant clinical milestones, functional assessment scales, and biochemical or imaging biomarkers to monitor disease evolution.

Recurring publications related to Mitochondrial Encephalomyopathy, Lactic Acidosis, and Stroke-like episodes (MELAS); Leigh syndrome; Leber’s Hereditary Optic Neuropathy (LHON); and Pearson syndrome were particularly informative. They supported the development of a preliminary list of 382 composite variables, considered relevant for establishing a structured data collection tool.

#### 2.1.3. Literature Synthesis

The data extracted from the reviewed literature emphasize the high degree of clinical and biological variability observed in mitochondrial cytopathies (MCs), both in terms of phenotypic expression and genotype–phenotype correlations [5,22,23,24]. This heterogeneity highlights the need for a carefully structured approach to data collection in order to meet diagnostic, epidemiological, and therapeutic objectives.

Moreover, very few studies, particularly at a multicenter or national level, propose a standardized framework for the elements to be collected. To our knowledge, no formally validated Minimum Data Set (MDS) currently exists for pediatric mitochondrial diseases. Some initiatives do exist, especially in the context of cohort studies or national registries such as the North American Mitochondrial Disease Consortium (NAMDC) in the United States or mitoNET in Germany, but none of them have reached a true expert consensus or undergone formal validation [25,26].

### 2.2. Identification of Key Variables: Delphi Method

#### 2.2.1. Rationale for the Chosen Method

The Delphi method was selected to establish expert consensus on the variables to be included in the Minimum Data Set (MDS) for pediatric mitochondrial cytopathies. This collaborative approach is particularly relevant when empirical data alone are insufficient and clinical practices vary across contexts—situations that are frequently encountered in the field of rare diseases [27,28].

The method allows for the structured and iterative consultation of a panel of experts through several anonymized rounds, free from hierarchical pressure or groupthink. This facilitates the emergence of a shared vision and genuine consensus [21].

#### 2.2.2. Expert Panel Composition

A panel of 16 experts with diverse professional backgrounds, statuses, and areas of practice was assembled, in accordance with international recommendations, which suggest involving between 10 and 50 experts to ensure both a broad spectrum of opinions and the methodological robustness of the process [29,30]. Previous studies in the field of rare diseases or MDS development have used panels of similar sizes, typically ranging from 15 to 40 participants [31].

Experts were selected based on the following inclusion criteria:

- A minimum of five years of professional experience in one or more of the following areas: biological diagnosis, clinical management, or research related to mitochondrial cytopathies;
- Institutional affiliation with a hospital, university, or public health organization;
- Availability to participate in both rounds of consultation, with prior informed consent.

The panel included neuropediatricians, general pediatricians, neurologists, molecular biologists, biochemists, public health researchers, and methodologists that were recruited from various hospital and academic institutions in Morocco and Tunisia.

#### 2.2.3. Questionnaire Development

A structured electronic questionnaire, incorporating the 382 composite variables identified during the narrative literature review, was developed and distributed to the expert panel in French to facilitate participation by all Maghreb experts involved in the study. Each question grouped together variables considered as potentially essential for inclusion in a Minimum Data Set (MDS).

Experts were asked to evaluate the relevance of each group of variables using a 5-point Likert scale:

1 = Not at all important

2 = Slightly important

3 = Moderately important

4 = Important

5 = Very important

An open-ended comment box was also included at the end of each question, allowing participants to justify their ratings and to suggest additional variables where relevant.

#### 2.2.4. Selection Criteria and Delphi Rounds Process

The Delphi process was carried out in two successive rounds. Sixteen participants were invited to complete both rounds. The final list of MDS variables was obtained across these two cycles. The first Delphi round lasted six weeks, and the second round lasted one week.

The content validity of the Delphi questionnaire was confirmed by a validation panel composed of neuropediatric specialists and two public health methodology researchers.

The results of the first round were analyzed according to the following criteria:

- Retained items: Variables rated 4 or 5 by more than 75% of experts were considered to have reached consensus and were included in the final version of the MDS.
- Items for re-evaluation: Variables with agreement between 60% and 75% were submitted for re-assessment in the second Delphi round.
- Rejected items: Variables rated 4 or 5 by fewer than 60% of experts were excluded from the questionnaire [32].

Between the two rounds, a summary of anonymized results was shared with participants, allowing them to reconsider their responses in light of the group’s overall evaluation, in keeping with the standard principles of the Delphi process.

### 2.3. Biostatistical Analysis and Content Validity Assessment

Data were analyzed using descriptive statistics in the open-access software JAMOVI 2.6.44.

The purpose of content validity is to evaluate the extent to which the elements of a measurement instrument appropriately and representatively reflect the conceptual domain they are intended to cover. In this study, we assessed the content validity of the composite questions derived from the Delphi consensus process, rather than each individual variable separately. This approach was chosen to reduce the cognitive burden on experts and to ensure maximum domain coverage.

This methodological choice is supported by Zamanzadeh et al. (2015) and Yaghmale (2009), who recommend such adaptations in content validity analyses when items are grouped into logical blocks [33,34].

This strategy also aligns with methodological recommendations for Delphi studies conducted in complex contexts, particularly those involving multiple dimensions and a limited number of expert participants [21,30,32].

#### 2.3.1. Content Validity Index

The Content Validity Index (CVI) represents the proportion of experts who rated each composite question as 4 (relevant) or 5 (very relevant) on a 5-point Likert scale.

Formula used is

CVI = (Number of experts rating ≥ 4)/(Total number of experts)

For panels of 10 or more experts, a minimum acceptable CVI of 0.78 is applied, following the recommendations of Lynn (1986) [35], in order to reduce the risk of false positives in content relevance assessment.

Two forms of CVI were calculated:

S-CVI/UA (Universal Agreement): the proportion of items for which all experts gave a rating of ≥4.

S-CVI/UA = Number of composite questions with full agreement/Total number of composite questions

S-CVI/Ave (Average CVI): the average of I-CVIs across all composite questions. It is a more flexible index than S-CVI/UA as it does not require full agreement.

S-CVI/Ave = ∑ I-CVI of all composite questions/Total number of composite questions

#### 2.3.2. Modified Kappa Coefficient

A modified Kappa coefficient (κ)* was calculated for each composite question to account for the potential chance agreement among experts, using the formula proposed by Polit, Beck, and Owen (2007) [36,37]:

Kappa = (I-CVI − Pₐ)/(1 − Pₐ)

where Pₐ is the probability of chance agreement.

Kappa values were interpreted based on the scale from Cicchetti and Sparrow (1981) [38]:

≥0.74: Excellent content validity
0.60–0.73: Good content validity
0.40–0.59: Fair content validity (acceptable but debatable)
<0.40: Poor content validity

#### 2.3.3. Content Validity Ratio

The Content Validity Ratio (CVR) was used to assess expert judgment on the essential nature of each composite question, using the formula

CVR = (nₑ − N/2)/(N/2)

where

n_e_ = number of experts who rated the item as essential;
N = total number of experts.

According to Lawshe’s table (1975) [39], the minimum acceptable CVR value was set at 0.33. Items with a CVR below this threshold were considered non-essential and excluded.

### 2.4. Ethical Considerations

The Morocco–Tunisia collaborative project titled “Clinical, Biochemical, and Molecular Investigations of Mitochondrial Cytopathies in Children” was submitted to the Research Ethics Committee of the Faculty of Medicine and Pharmacy of Rabat, Morocco, and ethical approval was obtained (Approval Number: CERB 17/22) on 28 March 2022.

Informed consent was obtained from all participants. They were assured of the confidentiality of their personal and professional identities throughout the study.

## 3. Results

### 3.1. Literature Review

The searches conducted in PubMed, Scopus, ScienceDirect, and Google Scholar led to the identification and selection of articles published within the North American Mitochondrial Disease Consortium (NAMDC), which remains one of the most widely used reference sources in the field [40]. Active since 2010, NAMDC includes data on over 1800 patients diagnosed with a mitochondrial cytopathy and serves as both a clinical database and a resource for multicenter clinical trials and translational research.

Another significant example is the Italian Registry of Patients with Mitochondrial Diseases (MITOCON) [41], which also includes neuropediatric data. As of 2023, the registry had enrolled 761 patients. It serves as a key instrument for scientific research and natural history studies of mitochondrial diseases, while also supporting clinical trials and the development of new therapeutic approaches. The registry contributes to international initiatives such as the GENOMIT project, which aims to harmonize national registries and foster collaborative networks across countries.

Based on the structure and data organization of NAMDC and MITOCON, as reported in associated scientific publications, five major data domains were identified (see Table 1):

Sociodemographic Data: age, sex, ethnic background, country of residence, etc.

General Clinical Data: family history, age at symptom onset, neurological, gastrointestinal, ocular symptoms, etc.

Biochemical Data: lactate, pyruvate, creatine phosphokinase (CPK), etc.

Molecular and Genetic Data: mitochondrial or nuclear DNA variants, sequencing methods, etc.

Treatment and Follow-Up Data: supplements used, experimental therapies, hospitalizations, etc.

### 3.2. Design of the Proposed MDS Model

An exhaustive and detailed model was developed, comprising 5 sections, 22 categories, 59 subcategories, and a total of 382 variables. This structure was established through multiple consultations with experts and key stakeholders in the field of mitochondrial cytopathies. These discussions enabled the identification of relevant data elements and specific variables to be included in the proposed MDS model (Table 2), ensuring alignment with the current needs and data collection practices of our clinical and research teams.

### 3.3. Delphi Survey

For this multicenter study, the expert panel was binational, comprising professionals from Morocco and Tunisia. It included 16 specialists from various fields: 7 pediatric neurologists, 2 medical geneticists, 3 biochemists, 1 neurologist, 2 pediatricians, and 1 public health methodologist. A total of 94% of the experts were women, with a mean age of 43.7 years ± 10.1, and 82% had more than 5 years of professional experience in mitochondrial cytopathies (Table 3).

To this end, a two-round Delphi survey was conducted to better identify the essential components to be included in a pediatric Minimum Data Set (MDS) for mitochondrial cytopathies. During the Delphi phase, a questionnaire consisting of 57 composite questions was submitted to the expert panel.

### 3.4. Evaluation of the MDS

#### 3.4.1. Content Validity Index

The Delphi process, conducted with a multidisciplinary panel of experts, enabled the assessment of the relevance of 382 initially proposed variables to develop a pediatric MDS for MCs. Results from both survey rounds revealed a strong convergence of expert opinions, with a high average relevance score (4.5 ± 0.41) on a 0-to-5 scale and an overall validation rate of 90.1% (347 variables retained).

Several sections achieved excellent acceptability levels, notably clinical data (mean: 4.54 ± 0.39), biochemical and molecular data (mean: 4.50 ± 0.33), and medical follow-up information (mean: 4.63 ± 0.39), with retention rates exceeding 85% of the proposed variables. Subcategories such as family history, specific neurological signs, radiological abnormalities, and neurophysiological assessments also showed strong consensus (CVI > 0.90).

Conversely, some variables related to less specific multisystemic manifestations (cutaneous, hematological, hepatic, or endocrine involvement) were rejected or only partially retained due to low perceived relevance (CVI < 0.60), low average scores, or low approval rates (≤75% of ratings ≥ 4). This was also the case for certain therapeutic parameters, such as transfusions and electrolyte supplementation, highlighting variability in practices depending on national clinical contexts (Table 4).

These findings reflect both the methodological rigor of the selection process and the aim of developing a pragmatic, streamlined, yet representative MDS that captures the complexity of pediatric MCs. The high inter-rater agreement (CVI and Kappa) supports the validity of the achieved consensus. As such, the 347 validated variables can serve as a foundational framework for establishing a bi-national (Morocco–Tunisia) registry to enhance monitoring, research, and care of these rare diseases.

#### 3.4.2. Calculation of Kappa and CVR Indices

To ensure methodological rigor and minimize potential biases, a statistical evaluation of each composite question was conducted through the calculation of the Kappa coefficient and the CVR. These analyses were applied to all questions across the five sections.

Based on the context of this study, the results corresponding to the five sections are presented in Table 5. Following the second round of the Delphi process, the analysis revealed that only one subcategory comprising five variables from the Therapeutic Data section was considered unnecessary by the experts. These five variables were excluded due to a Kappa value that fell below the accepted threshold for validity.

As a result of this validation process, the proposed MDS was refined to retain only the items deemed relevant and demonstrating excellent content validity, leading to a final version composed of 46 sub-classes and 347 variables.

#### 3.4.3. S-CVI

Table 6 presents the content validity results for each section of the MDS using the S-CVI/UA and S-CVI/Ave indices to express the degree of expert consensus on the relevance of the variables underlying each Delphi subset.

Overall, the five evaluated sections show relatively high S-CVI/Ave values, ranging from 0.91 to 0.99, reflecting a strong general agreement on the relevance of the retained items. The paraclinical data section showed the highest result (0.99), indicating excellent consistency in expert judgments. In contrast, the evolutionary data section, while still within acceptable limits (0.91), recorded the lowest score among the content validity levels generally recommended in the literature (threshold > 0.90).

Regarding the S-CVI/UA, which measures overall expert agreement for each item, the values varied between 0 (evolutionary data) and 0.83 (paraclinical data). Thus, several highly relevant elements such as the clinical data section, which contains numerous questions, often encountered challenges in achieving unanimous agreement among experts. This section recorded a particularly low S-CVI/UA of 0.36, highlighting the diversity of opinions concerning this complex domain.

These various indices reveal strong consensus across most sections while also providing detailed insights throughout the Delphi process into the more debated sections, particularly the evolutionary items and the criteria used to retain or exclude certain variables in the final version of the MDS.

### 3.5. Final MDS

At the end of this Delphi process, the number of variables was reduced from 382 to 347, with 38 variables excluded across 9 subclasses. Among these, 23 variables belonged to the clinical section and 15 variables to the therapeutic section. The final MDS therefore comprises 347 variables distributed across 5 sections, 21 classes, and 46 subclasses.

### 3.6. Registry Design

To identify expectations, priorities, and constraints related to the design of a binational pediatric MC registry, a section was included to collect expert opinions.

The responses show strong consensus in favor of establishing a structured, interoperable registry that is useful for clinical practice, research, and public health management.

Key barriers identified include limited access to advanced diagnostic tools (reported by 81% of experts) and low institutional recognition of rare diseases (reported by 66% of respondents). These findings justify the creation of a registry to improve the structuring, coordination, and standardization of patient care.

The expected priority functions involve data centralization and harmonization, integration of a longitudinal follow-up module (96% agreement), and networking of Maghreb regional stakeholders (62%). Most experts recommend developing a centralized, online, specialized, and secure digital platform capable of multi-criteria search functions (87%), conditional data sharing (75%), and automated report generation. This choice confirms the ambition for a dynamic and scalable tool that meets both clinical and research needs.

Project feasibility is nuanced by the identification of logistical (62%), financial (56%), and technical (50%) constraints, which are perceived as real but manageable. Experts suggest primarily public or mixed funding (56%), complemented by support from non-profit or scientific organizations. Exclusively private funding is widely rejected, reflecting a desire for transparency and scientific independence.

From an ethical standpoint, respondents emphasize strong data protection requirements: encryption (69%), informed consent (56%), restricted data access (69%), and sharing limited to approved research projects (87%). These elements necessitate a strict regulatory framework aligned with international standards for health data management.

Finally, experts identify several success indicators for the registry. Scientifically, these include publication outputs, integration into clinical trials, and discovery of new region-specific variants. Clinically, improvements in diagnostic rates and delays, as well as the introduction of standardized protocols, are major objectives. Economically and socially, experts highlight reduced costs related to diagnostic errors, better access to specialized care, and improved equity among patients.

Together, these results highlight a clear, coherent, and realistic vision put forth by experts for the design of a regional mitochondrial cytopathy registry. They lay the foundation for a structuring tool serving quality of care, scientific research, and public health policy in the field of rare diseases (Table 7).

## 4. Discussion

Pediatric MCs, although rare and heterogeneous, require a structured and harmonized approach for the collection of clinical, biological, and genetic data to better understand their natural history, optimize management, and support translational research. In this context, the development of an MDS represents a crucial step toward structuring a dedicated pediatric registry for these disorders.

The results of our study confirm the interest and feasibility of such an MDS, validated by a binational panel of Moroccan and Tunisian experts. The rigorous validation process, based on the Delphi method combined with quantitative analyses using CVI, Kappa, and CVR indices, enabled the establishment of a robust and consensual model comprising 347 variables distributed over 5 sections, 21 classes, and 46 subclasses. These findings are consistent with the international literature.

Indeed, several international registries have demonstrated the importance of finely structured data. The American NAMDC registry, active since 2010, serves as a reference model with over 1800 enrolled patients. This registry uses a structured dataset divided into five major categories—sociodemographic, clinical, biochemical, molecular, and therapeutic data—which are comparable to the sections retained in our MDS [42]. Similarly, the Italian MITOCON registry, which included 761 patients in 2023, relies on a similar organization and is integrated into international initiatives such as GENOMIT, promoting registry harmonization across Europe.

Our MDS model distinguishes itself by its pediatric focus and its capacity to address the clinical realities of Southern countries, taking into account local practices and resources. The high relevance of retained variables (mean CVI score: 4.5 ± 0.41) reflects a strong consensus, particularly in clinical domains (4.54 ± 0.39), biochemical and molecular fields (4.50 ± 0.33), and medical follow-up (4.63 ± 0.39). This outcome aligns with priorities identified in the literature, emphasizing the need for comprehensive documentation of multisystemic manifestations, biomarkers, and molecular abnormalities [43].

The identification of particularly strong consensus for neurological, radiological, and neurophysiological items aligns with the clinical profile of mitochondrial cytopathies, which predominantly affect the central nervous system in children [4]. Conversely, the lower validation of nonspecific involvements (cutaneous, endocrine, hepatic) reflects expert caution regarding their discriminative value, corroborating other studies on the phenotypic variability of these disorders [44].

The low S-CVI/UA score observed in the clinical section (0.36), despite a high average relevance, highlights a major methodological challenge: heterogeneity in clinical practices and the influence of local contexts. This finding echoes prior multicenter studies reporting divergences in how symptoms are identified, categorized, and prioritized according to medical cultures.

The validation approach adopted in this study, combining several content validity indices (I-CVI, S-CVI, Kappa, CVR), reinforces the robustness of the MDS. Previous methodological studies recommend this triangulation of indices to maximize the reliability of health-related MDSs [34,45].

The exclusion of 38 variables, mainly from the clinical and therapeutic sections, may seem restrictive but is justified by an optimization goal. Indeed, an MDS must be both comprehensive and pragmatic to be easily usable in resource-limited settings without compromising data quality. The final version of 347 variables thus appears as a reasonable compromise between completeness and applicability.

Finally, the establishment of a binational registry based on this MDS will strengthen regional cooperation in North Africa, enhance the visibility of local data in international collaborations, and promote equity in rare disease research. Harmonizing data formats will also allow interoperability with European and North American registries, thereby facilitating participation in international clinical trials.

**Limitations and Perspectives**

Among the study limitations, it is important to note the absence of external validation of the proposed MDS with a panel of European or other African experts, which could have further expanded the consensus scope.

Our literature review specifically targeted well-characterized mitochondrial syndromes, such as MELAS, Pearson syndrome, and Leigh syndrome. While this focus allowed for a detailed and clinically relevant dataset tailored to pediatric MCs frequently encountered in our region, it inherently limits the generalizability of the MDS to the broader spectrum of mitochondrial disorders. Given that mitochondrial diseases encompass hundreds of distinct syndromes with diverse clinical and molecular features, it is possible that some relevant variables pertaining to less common or newly described syndromes were not captured. This limitation underscores the necessity for ongoing updates and expansions of the MDS as new evidence and disease entities emerge, ensuring its continued relevance and applicability across the full mitochondrial disease spectrum.

Furthermore, the MDS remains an evolving tool, intended to be regularly updated in light of diagnostic, therapeutic, and genetic advances.

In this context, certain clinically important items, such as chronic intestinal pseudo-obstruction and the need for transfusion, did not meet the predefined consensus thresholds during the Delphi process and were excluded from the final core set. Nevertheless, these variables are preserved in the extended list and could be reintroduced in future iterations, particularly if pilot-phase validation confirms their relevance for specific subtypes such as Pearson syndrome or gastrointestinal motility disorders.

Finally, an essential next step will be to test the feasibility of data collection in real-world conditions through a pilot study in partner clinical centers, to evaluate robustness, ergonomics, and data completeness. This approach will help identify implementation barriers (workload, missing data, ethical constraints) and effectively address them before wider deployment.

From the perspective of large-scale application, the extensive nature of the MDS, with its 347 variables, may pose challenges in terms of data entry and completeness, particularly in the context of rare diseases and resource-limited settings. This reality highlights the need to develop practical implementation strategies to ensure optimal and sustainable use of the tool.

To facilitate its adoption, a multi-level structure is envisaged, distinguishing the essential variables (core base), required for a minimal data set, from optional variables (extended), intended for more comprehensive collection when resources allow. This approach aims to provide flexibility adapted to the constraints and priorities of each participating center.

Furthermore, a modular application of the MDS could enable progressive deployment by section or theme (for example: clinical, genetic, therapeutic), according to the specific objectives of the registry concerned (epidemiological surveillance, translational research, etc.).

Future implementation could also benefit from digital solutions, such as the development of dynamic electronic forms incorporating conditional logic, autofill functions, and decision support systems. These tools would help simplify data entry and reduce the burden on clinicians.

In addition, the creation of training materials and user guides is planned to support healthcare professionals in using the MDS, while promoting harmonized data collection practices.

Together, these perspectives, combined with the planned pilot study to assess field applicability, will contribute to making the MDS a pragmatic, scalable, and interoperable tool that is capable of meeting the demands of clinical research while remaining feasible in real-world conditions.

## 5. Conclusions

This work enabled the development and validation, through a rigorous and participatory methodology, of a structured, relevant, and context-adapted MDS for the management of pediatric MCs in the Maghreb region. By leveraging the expertise of clinicians, biologists, and researchers from Morocco and Tunisia, we reached a consensus on 347 variables deemed essential for the systematic documentation of clinical, paraclinical, biochemical, molecular, and therapeutic follow-up aspects of these rare and complex disorders.

This MDS represents far more than a standardized data collection tool: it provides a methodological foundation for establishing a binational registry, promoting data harmonization, consolidating research efforts, and improving the care of affected children. It will enable better characterization of regional clinical profiles, assessment of the most frequent mitochondrial respiratory chain deficiencies, and mapping of associated genetic mutations in North African populations.

This work also contributes to a broader dynamic of scientific equity and local capacity building in the field of rare diseases. It paves the way for future interoperability with existing international registries, thereby facilitating the participation of Southern countries in multicenter studies and clinical trials.

The project’s perspectives are diverse: launching the registry on a secure online platform, extending the MDS to adult forms and various hospital settings, integrating modules focused on quality of life, and exploiting the data for clinical, epidemiological, and translational research. This work serves as a foundation for more inclusive, collaborative, and representative precision medicine that respects the diversity of geographic and clinical contexts.

## Figures and Tables

**Table 1:** Data elements identified from the literature.

Sections	Variables
Sociodemographic Data	Age, sex, ethnic origin, family history
General Clinical Data	Developmental delay, regression, ataxia, dystonia, seizures, myopathy, brain imaging, multisystem involvement
Biochemical Data	Enzymatic deficiencies (Complexes I–V), lactate, CoQ10, PDH, other biomarkers (FGF21, GDF15, specific amino acids, acylcarnitine profiles)
Molecular and Genetic Data	mtDNA mutations, nDNA mutations, targeted genes
Treatment and Follow-Up Data	Medications used, dosage and frequency, treatment duration, treatment response, non-pharmacological interventions, etc.

**Table 2:** Proposed structure of the pediatric MC MDS.

Section	Category	Subcategory	Variables
Administrative Data	Demographic Data		Full name, sex, age, date of birth, region and city of residence, address, mother’s and father’s phone numbers
	Medical Follow-Up		Specialty of the center, department, consultation frequency, admission date, referring physician, hospital file number, medical coverage, registry entry date, age at symptom onset, age of suspicion, age of confirmation of diagnosis
Clinical Data	Family history data	Parental Data	Geographic origin of the father, geographic origin of the mother, parental consanguinity (Yes/No), degree of consanguinity (1st, 2nd, 3rd degree, distant), parental age at birth (maternal and paternal). similar case(s) in the family (relationship to index case, confirmed or suspected, clinical presentation) and deceased relative(s) (relationship to index case, age at death, cause of death)
		IUFD History	History of IUFD in siblings or close relatives (Yes/No), number of cases, gestational age at death, known cause, family relation to index patient.
		Neurological/Psychiatric History	Infantile encephalopathy, intellectual disability, autism spectrum disorders, behavioral disorders, cognitive decline, epilepsy, febrile seizures, gait disturbances, abnormal movements, hearing loss, visual impairments
		Non-neurological Medical History	Hepatic disorders, cardiac disorders, renal disorders, hematological disorders, endocrine disorders, digestive disorders
	Personal Medical History	Antenatal History	IUGR, congenital malformations
		Perinatal History	Term of birth, birth measurements (head circumference, weight, length), prematurity, post-term birth, perinatal distress, neonatal respiratory distress, neonatal jaundice, neonatal hypoglycemia, microcephaly, macrocephaly, low birth weight
		Postnatal History	Medical or surgical history
		Developmental History	Normal, primary psychomotor delay, delay with superimposed regression, regression following normal developmental history. Age at motor and language milestones, age at regression onset
		Onset Mode	Progressive, acute
		Triggering Factors	Infectious context, vaccination, recent head trauma, anesthesia, surgical intervention
	Initial Clinical Signs	Neurological Signs	Psychomotor delay, cranial nerve involvement, epileptic seizures (type, frequency), movement disorders (dystonia, ataxia), progressive encephalopathy
		Muscular Signs	Proximal muscle weakness, severe hypotonia, exercise intolerance
		Multisystemic Signs	Cardiomyopathy (type, severity), tubular renal insufficiency, hepatic involvement (liver failure, cytolysis), diabetes associated with hearing loss, gastrointestinal involvement
		Other Specific Symptoms	Visual disturbances (optic neuropathy), sensorineural hearing loss, recurrent lactic acidosis, stunted statural and ponderal growth
	Central Nervous System Involvement	Specific Clinical Signs	Intellectual disability, behavioral disorders, epileptic seizures, pseudo-encephalitic episode(s), consciousness disturbances, stroke-like episode(s), gait disturbances, abnormal movements: dystonia, chorea, myoclonus, tremor, parkinsonism, rigidity, hypokinesia, others, pyramidal syndrome, axial hypotonia, spasticity, cerebellar ataxia, central cranial nerve involvement, microcephaly (<−2 SD), macrocephaly (>+2 SD)
		Medical Examinations	Neuropsychological, speech/language, and motor evaluations.
		Radiological Examinations	Cerebral CT scan, brain and spinal MRI, and magnetic resonance spectroscopy
		Interpretation and Type of Abnormality	Signal abnormalities in the basal ganglia, brainstem signal abnormalities, leukodystrophy, stroke-like lesions, cerebellar atrophy, cerebral calcifications, and lactate peak on spectroscopy.
		EEG/Interpretation and Types of Abnormalities:	Background rhythm abnormalities, epileptic discharges (focal, generalized, hypsarrhythmia), cortical myoclonus, subcortical myoclonus, and photosensitivity.
	Neuromuscular Involvement	Associated Syndromes and Symptoms	Peripheral neurogenic syndrome, myogenic syndrome, fatigability/exercise intolerance, rhabdomyolysis, ptosis, and ophthalmoplegia/paresis.
		Biological Workup	CPK, LDH, AST, ALT
		EMG	Normal, neurogenic involvement, polyneuropathy, polyradiculoneuropathy, sensory neuropathy, motor neuropathy, sensorimotor neuropathy, axonal, demyelinating, axonodemyelinating, signs of denervation, and myogenic involvement.
	Sensory Involvement	Type of Impairment	Hearing loss, DVA.
		Ophthalmologic Examination	Normal, optic atrophy, retinitis pigmentosa, cherry-red spot, cataract.
		AEP	Results: normal, sensorineural hearing loss (unilateral or bilateral), and auditory threshold.
		VEP	Results: normal; unilateral or bilateral optic neuropathy; axonal (reduced amplitude); demyelinating (prolonged latency); axonodemyelinating; absence of recordable potentials.
		Other Examinations	Results of the following assessments: visual field testing, OCT, ERG.
		Dysmorphology	Presence/absence; Facial features: hypertelorism, prominent forehead, cleft palate or lip, low-set ears, micrognathia; Body features: camptodactyly, spinal deformity (scoliosis), pectus excavatum, trident hand; Severity: mild/moderate/severe. Clinical photographs with consent.
	Skin involvement	Disorders	Hypertrichosis, eczema, alopecia, multiple lipomatosis, pruritic scaly erythema, reticular pigmentation, and vitiligo.
	Renal involvement	Disorders	Polyuria–polydipsia syndrome, low urine specific gravity (<1010), proteinuria detected by dipstick, urine acidification disorder (urinary pH > 5.5 in acidosis), and leukocyturia; tubulopathy, microalbuminuria, interstitial nephropathy, nephrotic syndrome, renal failure, nephrocalcinosis, and kidney stones (nephrolithiasis).
		Renal Laboratory Tests	Phosphaturia, calciuria, serum phosphate, serum calcium, serum sodium, FeNa, serum potassium, FeK, serum uric acid, and fractional excretion of uric acid.
	Liver involvement	Disorders	Jaundice, hemorrhagic syndrome, edema syndrome, hepatomegaly, splenomegaly, ascites, portal hypertension, and liver failure.
		Abdominal Ultrasound	Hepatomegaly, splenomegaly, hepatic steatosis, ascites, renal calcifications (nephrocalcinosis), digestive or urological malformations, abdominal effusions.
		Liver function tests	Cytolysis, AST, ALT, biochemical cholestasis, ALP, AFP.
	Cardiovascular involvement	Disorders	Signs of heart failure, HTN, palpitations, atrioventricular block, atrial fibrillation, WPW syndrome, and long QT syndrome.
		Cardiovascular Assessments	ECG data; TTE; HCM; signs of obstruction; DCM; mixed cardiomyopathy; LVNC; LVEF; and Holter monitoring.
	Hematological involvement	CBC	unexplained anemia, neutropenia, leukopenia, thrombocytopenia, pancytopenia
		Bone Marrow Examination	Integration of bone marrow findings, evaluation for dyserythropoiesis, sideroblastic anemia.
	Endocrine involvement	Growth Delay	Data on bone age, chronological age, and stature.
		Disorders	Growth retardation (in height or weight), presence of micropenis, or episodes of hypoglycemia; Diabetes, hypogonadism, adrenal insufficiency (central vs. peripheral).
		Endocrine Laboratory Tests	GH stimulation, IGF-1, and hormonal assays such as cortisol and TSH.
	Gastrointestinal involvement	Disorders	Chronic diarrhea, recurrent vomiting, or steatorrhea (frequency, duration, or triggering factors), Chronic intestinal pseudo-obstruction, exocrine pancreatic insufficiency, villous atrophy.
		Biological Workup	Fecal elastase, serum amylase, serum lipase
Paraclinical Data	Biochemical study	Initial Workup	Blood gas analysis, lactatemia, redox ratio (blood and CSF), redox cycle, UOA and POA.
		Confirmation Workup	Muscle biopsy under light microscopy, muscle biopsy under electron microscopy, and analysis of respiratory chain complexes (I to IV) on muscle biopsy.
	Molecular Study	Performed in:	Index case, parents (mother/father), other family members.
		Technique	Sanger sequencing, NGS (nuclear gene panel, mitochondrial gene panel, WES, WGS)
		Genetic Variants	Classification (pathogenic, likely pathogenic, uncertain significance), suspected gene, mitochondrial DNA deletions (mtDNA deletions).
		Molecular Diagnostic Status	Molecular diagnosis confirmed or not confirmed.
Therapeutic Data	Pharmacological treatment	Energy Treatment	Coenzyme Q10, L-carnitine, thiamine, biotin, riboflavin, arginine.
		Antiepileptic Treatment	Antiepileptic drugs (AEDs) such as Sodium Valproate, Carbamazepine, Phenobarbital, and others are commonly used. Treatment of abnormal movements: Medications including L-Dopa, Tetrabenazine, Trihexyphenidyl, etc., are used to manage movement disorders.
		Hormone Replacement Therapy	GH, hydrocortisone, thyroid hormones, and insulin
		Electrolyte Supplementation	Potassium, sodium, calcium, phosphorus, bicarbonate.
		Other Treatments	Antiemetics, pancreatic enzymes, domperidone, AUDC, vitamins K and D, iron, folate.
		Transfusions	RBC, platelets.
	Non-pharmacological treatment	Supportive Treatments	Physiotherapy (respiratory or motor), speech therapy rehabilitation, non-invasive ventilation, deep brain stimulation.
Evolution Data	Follow-up		Patient status: still under follow-up, lost to follow-up, deceased.
	Complications		Bedridden state, tendon retractions, fractures, hip dislocation, scoliosis, pressure ulcers (bedsores), malnutrition/cachexia.
	Overall evolution		Complete improvement, near-complete improvement (minimal symptoms), partial improvement, stable condition, deterioration, fluctuating course, death, fulminant form, slowly progressive form, benign form.

IUFD: Intrauterine fetal demise; IUGR: Intrauterine growth restriction; EMG: Electroneuromyography; DVA: Decreased visual acuity; VEP: Visual evoked potentials; OCT: Optical coherence tomography; ERG: Electroretinogram; FeNa: Fractional excretion of sodium; FeK: Fractional excretion of potassium; AST: Aspartate aminotransferase; ALT: Alanine aminotransferase; ALP: Alkaline phosphatase; AFP: Alpha-fetoprotein; HTN: Hypertension; WPW: Wolff–Parkinson–White syndrome; TTE: Transthoracic echocardiography; HCM: Hypertrophic cardiomyopathy; DCM: Dilated cardiomyopathy; LVNC: Left ventricular non-compaction; LVEF: Left ventricular ejection fraction; CBC: Complete blood count; GH: Growth hormone stimulation; IGF-1: Insulin-like growth factor 1; TSH: Thyroid-stimulating hormone; UOA: Organic acids in urine; POA: Organic acids in plasma; WES: Whole-exome Sequencing; WGS: Whole-genome Sequencing; GH: Growth hormone; RBC: Red blood cell concentrates.

**Table 3:** Demographic characteristics of the Delphi study participants.

Characteristics		Number of Participants	
		First Round N = 16	Second Round N = 10
Gender	Female	15 (94)	9 (90)
	Male	1 (6)	1 (10)
Age group (year)	30–40	7 (44)	2 (20)
	40–50	6 (38)	7 (70)
	50–60	1 (6)	1 (10)
	>60	2 (13)	**-**
Origin	Rabat	7 (44)	5 (50)
	Tanger	1 (6)	1 (10)
	Tunis	8 (50)	4 (40)
Affiliation	University Hospital	14 (88)	8 (80)
	Public Hospital	1 (6)	1 (10)
	Foundation	1 (6)	1 (10)
Medical specialty	Clinical Biochemistry	3 (19)	1 (10)
	Clinical Genetics	2 (13)	-
	Neurology	1 (6)	1 (10)
	Neuropediatrics	7 (44)	7 (70)
	General Pediatrics	2 (13)	1 (10)
	Public Health	1 (6)	-
Experience (years)-MCs	<5	3 (19)	-
	5–10	7 (44)	4 (40)
	>10	6 (38)	6 (60)
Frequency of management of MCs	Rarely (<5 cas/an)	6 (38)	1 (10)
	Occasionally (5–15 cas/an)	6 (38)	6 (60)
	Frequently (>15 cas/an)	4 (25)	3 (30)

**Table 4:** Summary of relevance scores and final selection of MDS variables.

Section	Category	Subcategory	N Variables	Score Mean (±ÉT)	Score Median	First Round Delphi % Scores ≥ 4	Second Round Delphi % Scores ≥ 4	CVI		Final Decision	N Final Variables
								Relevant (Score 4 or 5)	CVIs		
Administrative data	Demographic data		9	4.75 (±0.45)	5	100		16	1	Kept	9
	Medical follow-up		11	4.38 (±0.72)	4.5	87.5		14	0.87	Kept	11
Clinical data	Family history data	Parental data	11	4.63 (±0.50)	5	100		16	1	Kept	11
		IUFD history	5	4.13 (±1.31)	5	69	100	10	1	Kept	5
		Neurological/Psychiatric history	11	4.75 (±0.58)	5	94		15	0.94	Kept	11
		Non-neurological medical history	6	4.63 (±0.81)	5	94		15	0.94	Kept	6
	Personal medical history	Perinatal history	17	4.50 (±0.82)	5	94		15	0.94	Kept	17
		Developmental history	6	4.69 (±0.60)	5	94		15	0.94	Kept	6
		Onset mode/triggering factors	7	4.56 (±0.51)	5	100		16	1	Kept	7
	Initial clinical signs	Neurological signs	7	4.88 (±0.34)	5	100		16	1	Kept	7
		Muscular signs	3	4.88 (±0.34)	5	100		16	1	Kept	3
		Multisystemic signs	7	4.63 (±0.62)	5	94		15	0.94	Kept	7
		Other specific symptoms	4	4.50 (±1.03)	5	94		15	0.94	Kept	4
	Central Nervous system involvement	Specific clinical signs	22	4.81 (±0.40)	5	100		16	1	Kept	22
		Medical and radiological examinations	6	4.81 (±0.40)	5	100		16	1	Kept	6
		Interpretation and type of abnormalities	7	4.50 (±1.03)	5	94		15	0.94	Kept	7
		EEG/Interpretation and types of abnormalities	7	4.38 (±0.96)	5	82		13	0.82	Kept	7
	Neuromuscular involvement	Associated syndromes and symptoms	6	4.69 (±0.48)	5	100		16	1	Kept	6
		Biological workup	4	4.56 (±0.81)	5	94		15	0.94	Kept	4
		EMG	12	4.44 (±1.09)	5	88		14	0.88	Kept	12
	Sensory involvement	Type of impairment	2	4.75 (±0.45)	5	100		16	1	Kept	2
		Ophthalmologic examination	5	4.81 (±0.40)	5	100		16	1	Kept	5
		AEP	4	4.13 (±1.02)	4	88		14	0.88	Kept	4
		VEP	7	4.25 (±1.00)	4	94		15	0.94	Kept	7
		Other examinations	3	3.69 (±1.20)	4	56		9	0.56	Removed	0
		Dysmorphology	14	4.31 (±0.95)	5	81		13	0.81	Kept	14
	Skin involvement	Disorders	7	3.81 (±1.17)	4	57		9	0.57	Removed	0
	Renal involvement	Disorders	5 12	4.38 (±0.72)	4.5	88		14	0.88	Kept	12
		Renal laboratory tests	10	4 (±1.21)	4	75	100	10	1	Kept	10
	Liver involvement	Disorders	8	4.38 (±1.09)	5	88		14	0.88	Kept	8
		Abdominal ultrasound	7	3.81 (±1.22)	4	63	90	9	0.90	Kept	7
		Liver function tests	7	4.44 (±0.81)	5	94		15	0.94	Kept	7
	Cardiovascular involvement	Disorder	7	4.69 (±0.48)	5	100		16	1	Kept	7
		Cardiovascular assessments	9	3.75 (±1.29)	4	63	90	9	0.90	Kept	9
	Hematological involvement	CBC	5	4.38 (±1.02)	5	88		14	0.88	Kept	5
		Bone marrow examination	3	3.56 (±1.26)	3.5	50		8	0.5	Removed	0
	Endocrine involvement	Growth delay	3	4.25 (±1.13)	5	81		13	0.81	Kept	3
		Laboratory tests	4	3.56 (±1.41)	4	57		9	0.57	Removed	0
		Disorders	9	3.94 (±1.34)	4	75	100	10	1	Kept	9
	Gastrointestinal involvement	Disorders	5	4.19 (±1.11)	4.5	81		13	0.81	Kept	5
		Laboratory tests	3	3 (±1.37)	3	32		5	0.32	Removed	0
Paraclinical Data	Biochemical study	Initial workup	7	5 (±0.00)	5	100		16	1	Kept	7
		Confirmation workup	3	4.94 (±0.25)	5	100		16	1	Kept	3
	Molecular study	Performed in:	3	4.94 (±0.25)	5	100		16	1	Kept	3
		Technique	5	4.88 (±0.50)	5	94		15	0.94	Kept	5
		Genetic variants	5	4.94 (±0.25)	5	100		16	1	Kept	5
		Molecular diagnostic status	2	4.69 (±0.48)	5	100		16	1	Kept	2
Therapeutic Data	Pharmacological treatment	Energy treatment	6	4.69 (±0.48)	5	100		16	1	Kept	6
		Antiepileptic treatments	6	4.31 (±0.87)	5	75	90	9	0.90	Kept	6
		Hormone replacement therapy	4	4.19 (±1.11)	4.5	81		13	0.81	Kept	4
		Electrolyte supplementation	5	3.63 (±1.20)	4	63	10	1	0.1	Removed	0
		Other treatments	8	3.56 (±1.15)	3.50	50		8	0.5	Removed	0
		Transfusions	2	3.13 (±1.31)	3	37,6		6	0.37	Removed	0
	Non- pharmacological treatment	Supportive treatments	4	4.13 (±1.15)	4.50	75	100	10	1	Kept	4
Evolution Data	Follow-up		3	4.69 (±0.60)	5	94		15	0.94	Kept	3
	Complications		7	4.06 (±1.34)	5	75	90	9	0.90	Kept	7
	Overall evolution		10	4.50 (±1.10)	5	88		14	0.88	Kept	10
Total items		57	382								347

**Table 5:** Calculation of CVR, Kappa, and final interpretation.

Category	Subcategory	N Variables	Relevant (Score 4 or 5)	CVR	CVI (>=0.78)	Pc	K	Interpretation
Demographic data		9	16	1	1	0.00001526	1	Excellent content validity
Medical follow-up		11	14	0.75	0.87	0.00183	0.87	Excellent content validity
Family history data	Parental data	11	16	1	1	0.00001526	1	Excellent content validity
	IUFD history	5	10	1	1	0.0009765625	1	Excellent content validity
	Neurological/Psychiatric history	11	15	0.875	0.94	0.0002441	0.94	Excellent content validity
	Non-neurological medical history	6	15	0.875	0.94	0.0002441	0.94	Excellent content validity
Personal medical history	Perinatal history	17	15	0.875	0.94	0.0002441	0.94	Excellent content validity
	Developmental history	6	15	0.875	0.94	0.0002441	0.94	Excellent content validity
	Onset mode/triggering factors	7	16	1	1	0.00001526	1	Excellent content validity
Initial clinical signs	Neurological signs	7	16	1	1	0.00001526	1	Excellent content validity
	Muscular signs	3	16	1	1	0.00001526	1	Excellent content validity
	Multisystemic signs	7	15	0.875	0.94	0.0002441	0.94	Excellent content validity
	Other specific symptoms	4	15	0.875	0.94	0.0002441	0.94	Excellent content validity
Central Nervous system involvement	Specific clinical signs	22	16	1	1	0.00001526	1	Excellent content validity
	Medical and radiological examinations	6	16	1	1	0.00001526	1	Excellent content validity
	Interpretation and type of abnormalities	7	15	0.875	0.94	0.0002441	0.94	Excellent content validity
	EEG/Interpretation and types of abnormalities	7	13	0.625	0.82	0.00855	0.82	Excellent content validity
Neuromuscular involvement	Associated syndromes and symptoms	6	16	1	1	0.00001526	1	Excellent content validity
	Biological workup	4	15	0.875	0.94	0.0002441	0.94	Excellent content validity
	EMG	12	14	0.75	0.88	0.00183	0.88	Excellent content validity
Sensory involvement	Type of impairment	2	16	1	1	0.00001526	1	Excellent content validity
	Ophthalmologic examination	5	16	1	1	0.00001526	1	Excellent content validity
	AEP	4	14	0.75	0.88	0.00183	0.88	Excellent content validity
	VEP	7	15	0.875	0.94	0.0002441	0.94	Excellent content validity
	Dysmorphology	14	13	0.625	0.81	0.00855	0.81	Excellent content validity
Renal involvement	Disorders	5	14	0.75	0.88	0.00183	0.88	Excellent content validity
	Renal laboratory tests	10	10	1	1	0.0009765625	1	Excellent content validity
	Renal pathologies	7	13	0.625	0.81	0.00855	0.81	Excellent content validity
Liver involvement	Pathologies	8	14	0.75	0.88	0.00183	0.88	Excellent content validity
	Abdominal ultrasound	7	9	0.8	0.9	0.0002400	0.9	Excellent content validity
	Liver function tests	7	15	0.875	0.94	0.0002441	0.94	Excellent content validity
Cardiovascular involvement	Pathology or disorder	7	16	1	1	0.00001526	1	Excellent content validity
	Cardiovascular assessments	9	9	0.8	0.9	0.0002400	0.9	Excellent content validity
Hematological involvement	CBC	5	14	0.75	0.88	0.00183	0.88	Excellent content validity
Endocrine involvement	Growth delay	3	13	0.625	0.81	0.00855	0.81	Excellent content validity
	Disorders	4	15	0.875	0.94	0.0002441	0.94	Excellent content validity
	Pathologies	5	10	1	1	0.0009765625	1	Excellent content validity
Gastrointestinal involvement	Disorders	5	13	0.625	0.81	0.00855	0.81	Excellent content validity
Biochemical study	Initial workup	7	16	1	1	0.00001526	1	Excellent content validity
	Confirmation workup	3	16	1	1	0.00001526	1	Excellent content validity
Molecular study	Performed in	3	16	1	1	0.00001526	1	Excellent content validity
	Technique	5	15	0.875	0.94	0.0002441	0.94	Excellent content validity
	Genetic variants	5	16	1	1	0.00001526	1	Excellent content validity
	Molecular diagnostic status	2	16	1	1	0.00001526	1	Excellent content validity
Pharmacological treatment	Energy treatment	6	16	1	1	0.00001526	1	Excellent content validity
	Antiepileptic treatments	6	9	0.8	0.9	0.0002400	0.9	Excellent content validity
	Hormone replacement therapy	4	13	0.625	0.81	0.00855	0.81	Excellent content validity
Non- pharmacological treatment	Supportive treatments	4	10	1	1	0.0009765625	1	Excellent content validity
Follow-up		3	15	0.875	0.94	0.0002441	0.94	Excellent content validity
Complications		7	9	0.8	0.9	0.0002400	0.9	Excellent content validity
overall evolution		10	14	0.75	0.88	0.00183	0.88	Excellent content validity

**Table 6:** S-CVI by section.

Sections	N Variables	N composite Questions	S-CVI/UA	S-CVI/Ave
Administrative Data	20	2	0.5	0.93
Clinical Data	262	36	0.36	0.93
Paraclinical Data	25	6	0.83	0.99
Therapeutic Data	20	4	0.5	0.92
Evolution Data	20	3	0	0.91

**Table 7:** Expert consensus on the registry structure.

Expert Consensus				Frequency (%) N = 16
Registry structure	Challenges in managing mitochondrial cytopathies		Access to advanced diagnostic tools	13 (81)
			Limited recognition of rare diseases in health policies	3 (19)
	Priorities for the design of a mitochondrial registry		Centralize clinical and paraclinical data	13 (81)
			Standardize data collection and analysis methods	10 (62)
			Support collaboration across Maghreb medical centers	10 (62)
			Estimate the prevalence of the disease in Morocco	1 (6)
	Inclusion of a longitudinal patient follow-up module		Yes, it is essential	15 (96)
			No, cross-sectional data are sufficient	1 (6)
	Features to include in the registry		Multicriteria search	14 (87)
			Automatic report generation	8 (50)
			Secure data sharing among researchers	12 (75)
			Longitudinal patient follow-up	10 (62)
Technical and organizational aspects	Registry data format		Online digital platform	13 (81)
			Local database at each center with periodic synchronization	3 (19)
	Data structure		Multicenter standardization	2 (12)
			Continuous or periodic data collection	3 (19)
			Digital data format such as Excel file	1 (6)
			Digital data format of SQL database type	-
			Custom platforms	10 (62)
	Challenges in setting up a Maghreb registry		Technical aspects	8 (50)
			Financial aspects	9 (56)
			Logistical aspects	10 (62)
	Funding sources or partnerships to support the registry		Public funding	2 (12)
			Private funding	1 (6)
			Mixed funding (public and private)	9 (56)
			Scientific organizations	1 (6)
			Non-profit organizations	2 (12)
Security and ethics	Measures to ensure patient data confidentiality		Data encryption	11 (69)
			Systematic informed consent	9 (56)
			Restricted access to authorized personnel	11 (69)
	Integration of a data sharing policy to promote research		Yes, under strict conditions	14 (87)
			No, to preserve patient confidentiality	2 (12)
Projections and Impact Evaluation	Expected outcomes of establishing a dedicated registry for MCs	Scientific publications		15 (94)
		Improvement of diagnostic protocols		15 (94)
		New therapeutic strategies		14 (88)
		Strengthening of multicenter collaborations		15 (94)
	Recommended indicators to assess the success and impact of the registry in the medium and long term	Scientific indicators	Number of scientific publications based on registry data	13 (81)
			National and international collaborations initiated through the registry	15 (94)
			Identification of new mutations or specific Maghrebian genetic profiles	16 (100)
			Participation in clinical trials based on registry data	14 (87)
		Clinical indicators	Reduction of the average delay between symptom onset and diagnosis	14 (87)
			Improvement in the accuracy of diagnoses through registry data	13 (81)
			Implementation of new standardized diagnostic and therapeutic protocols	15 (94)
			Improvement in survival rates and quality of life of followed-up patients	14 (87)
		Registry management indicators	Total number of patients enrolled in the registry	10 (62)
			Data completeness	12 (75)
			Number of clinical centers or laboratories participating in the registry	12 (75)
			Frequency of registry data updating and analysis	13 (81)
		Economic indicators	Reduction of costs related to diagnostic errors or treatments	12 (75)
			Additional funding obtained through the registry	13 (81)
			Economic impact on healthcare systems	13 (81)
		Social indicators	Increased access to specialized care for patients with MCs	14 (87)
			Assessment of the impact on the quality of life of patients and their families	13 (81)
			Improvement of equity in patient care among different social classes	11 (69)

## Data Availability

To maintain the confidentiality of the participants and health services involved, the data presented in this study are available from the corresponding author upon request.

## Abbreviations

The following abbreviations are used in this manuscript:

AFP	Alpha-fetoprotein
AHRQ	Agency for healthcare research and quality
ALP	Alkaline phosphatase
ALT	Alanine aminotransferase
AST	Aspartate aminotransferase
CBC	Complete blood count
CMs	Mitochondrial cytopathies
CVI	Content Validity Index
CVR	Content Validity Ratio
DCM	Dilated cardiomyopathy
DVA	Decreased visual acuity
EMG	Electroneuromyography
ERG	Electroretinogram
FeK	Fractional excretion of potassium
FeNA	Fractional excretion of sodium
FGF21	Fibroblast growth factor 21
GH	Growth hormone stimulation
GDF15	Growth differentiation factor 15
HCM	Hypertrophic cardiomyopathy
HIMSS	Health Information and Management Systems Society
HTN	Hypertension
IGF-1	Insulin-like growth factor 1
IUFD	Intrauterine Fetal Demise
IUGR	Intrauterine growth restriction
LHON	Leber’s hereditary optic neuropathy
LVEF	Left ventricular ejection fraction
LVNC	Left ventricular non-compaction
MDS	Minimum Data Set
MELAS	Mitochondrial encephalopathy, lactic acidosis, and stroke-like episodes
MITOCON	Italian Registry of Patients with Mitochondrial Diseases
NAMDC	North American Mitochondrial Disease Consortium
OCT	Optical coherence tomography
POA	Organic acids in plasma
RBC	Red blood cell concentrates
S-CVI/Ave	Average of CVI across items
S-CVI/UA	Universal Agreement
TSH	Thyroid-stimulating hormone
TTE	Transthoracic echocardiography
UOA	Organic acids in urine
VEP	Visual evoked potentials
WES	Whole-exome sequencing
WGS	Whole-genome sequencing
WPW	Wolff–Parkinson–White syndrome
